# Connecting *SiO*_4_ in Silicate and Silicate Chain Networks to Compute Kulli Temperature Indices

**DOI:** 10.3390/molecules27217533

**Published:** 2022-11-03

**Authors:** Ying-Fang Zhang, Muhammad Usman Ghani, Faisal Sultan, Mustafa Inc, Murat Cancan

**Affiliations:** 1 School of Mathematics and Information Science, Henan Polytechnic University, Jiaozuo 454000, China; 2 Institute of Mathematics, Khawaja Fareed University of Engineering & Information Technology, Abu Dhabi Road, Rahim Yar Khan 64200, Pakistan; 3 Department of Mathematics, Firat University, 23119 Elazig, Turkey; 4 Department of Medical Research, China Medical University, Taichung 40402, Taiwan; 5 Faculty of Education, Yuzuncu Yil University, 65080 Van, Turkey

**Keywords:** temperature indices, silicate network, silicate chain network

## Abstract

A topological index is a numerical parameter that is derived mathematically from a graph structure. In chemical graph theory, these indices are used to quantify the chemical properties of chemical compounds. We compute the first and second temperature, hyper temperature indices, the sum connectivity temperature index, the product connectivity temperature index, the reciprocal product connectivity temperature index and the F temperature index of a molecular graph silicate network and silicate chain network. Furthermore, a QSPR study of the key topological indices is provided, and it is demonstrated that these topological indices are substantially linked with the physicochemical features of COVID-19 medicines. This theoretical method to find the temperature indices may help chemists and others in the pharmaceutical industry forecast the properties of silicate networks and silicate chain networks before trying.

## 1. Introduction

Using chemical graph theory, one can determine a wide range of characteristics, such as chemical networks, physical, chemical, and thermal properties, biological activity, and chemical activity [1]. Topological indices, which are molecular descriptors, can characterize these features and specific graphs [2,3]. In chemical graph theory, vertices represent atoms, and edges represent chemical bonding between the atoms [4,5]. The topological index of a chemical composition is a numerical value or a continuation of a given structure under discussion, which indicates the chemical, physical and biological properties of a structure of chemical molecule; see for details [6,7,8].

Mathematical chemistry explains how to use polynomials and functions to provide instructions hidden in the symmetry of molecular graphs, and graph theory has many applications in modern chemistry, particularly organic chemistry. Many applications of topological indices are used in theoretical chemistry, particularly QSPR/QSAR research. Many well-known researchers have investigated topological indices in order to learn more about various graph families [9]. In qualitative structure property relationships (QSPR) and qualitative structure activity relationships (QSAR), topological indices are used directly as simple numerical descriptors in comparison with physical, biological, or chemical parameters of molecules, which is an advantage of the chemical industry. Many researchers have worked on various chemical compounds and computed topological descriptors of various molecular graphs over the last few decades [10,11].

In a recent article [12], the atom-bond sum-connectivity (ABS) index was proposed as a new molecular descriptor by combining the key ideas of the SC and ABC indices. Graph indices have been discovered to be useful in chemistry for chemical documentation, structure property relationships, structure activity relationships, and pharmaceutical drug design. There has been much interest in the general issue of calculating graph indices [13,14].

We only consider finite, simple, connected graphs in this paper. Assume *G* is a graph with vertex set $V_{G}$ and edge set $E_{G}$; the number of vertices adjacent to a vertex *u* determines its degree $d_{u}$. For fundamental notations and terminologies, we refer the reader to [15].

Fajtlowicz defined the temperature of every vertex *u* of a graph *G* in [16] as

(1) $$T_{u_{i}}=\frac{d_{u_{i}}}{|V_{G}|\;-d_{u_{i}}}\;\;\;where\;\;\;\forall \;\;u_{i}\in V_{G}$$

The first temperature index [17] is defined as follows:

(2) $$T_{1}\left(G\right)=\sum_{u,v\in E(G)}\left(T_{u}+T_{v}\right)$$

In 2020, Kulli introduced the second temperature index [18], which is defined as follows:

(3) $$T_{2}\left(G\right)=\sum_{u,v\in E(G)}\left(T_{u}\times T_{v}\right)$$

Kulli introduced the first and second hyper temperature indices in [18], which are defined as

(4) $$HT_{1}\left(G\right)=\sum_{u,v\in E(G)}{\left(T_{u}+T_{v}\right)}^{2}$$

(5) $$HT_{2}\left(G\right)=\sum_{u,v\in E(G)}{\left(T_{u}\times T_{v}\right)}^{2}$$

Of note, also introduced in the same paper [18] were the sum connectivity temperature index, the product connectivity temperature index, and the reciprocal product connectivity index, which are defined as

(6) $$ST\left(G\right)=\sum_{u,v\in E(G)}\frac{1}{\sqrt{\left(T_{u}+T_{v}\right)}}$$

(7) $$PT\left(G\right)=\sum_{u,v\in E(G)}\frac{1}{\sqrt{\left(T_{u}\times T_{v}\right)}}$$

(8) $$RPT\left(G\right)=\sum_{u,v\in E(G)}\sqrt{\left(T_{u}\times T_{v}\right)}$$

Kulli introduced the F-temperature index and general temperature index of a graph G in [18], and they are defined as

(9) $$FT\left(G\right)=\sum_{u,v\in E(G)}\left(T_{u}^{2}+T_{v}^{2}\right)$$

In industrial chemistry, a silicate Si is an element of a family of anions (an ion is a atom or molecule with a net electrical charge) containing of silicon and oxygen. L. Boyer used the general formula ${\left[SiO{}_{4-t}^{(4-2t)-}\right]}_{n}$ for 0≤t<2 in [19]. Some researchers also explain the family of anions by using a formula for the orthosilicate family, $SiO_{4}^{4-}\left(t=0\right)$, as can be seen in [20]; a formula for the metasilicate family, $SiO_{3}^{2-}\left(t=1\right)$, as can be seen in [21]; and a formula for the pyrosilicate family, $Si_{2}O_{7}^{6-}\left(t=\frac{1}{2},n=2\right)$, as can be seen in [22]. We can extend silicate $S_{i}$ to any anion containing silicon (atom bonding with something other than $O_{2}$), such as Hexafluorosilicate $SiF_{3}^{2-}$; see in [23]. Here, we discuss only chains of silicates, which are obtained by alternating sequence of the tetrahedral $SiO_{4}$; see for details [24,25].

In this article, the above-defined eight temperature indices are constructed by the atom bond partition of a silicate network $SN_{P}$ and a silicate chain network $CN_{P}$, which are partitioned according to the degrees of their $S_{i}$ and $O_{2}$ atoms. We also investigate the silicon tetrahedron $S_{i}O_{4}$ in a compound structure and derive the precise formulas of certain essential degree-based temperate indices using the approach of atom bond partitioning of the molecular structure of silicates. We use the the concept of temperature indices from Kulli and other researchers [26,27].

## 2. Results for Silicate Network $SN_{P}$

**Figure 1:**
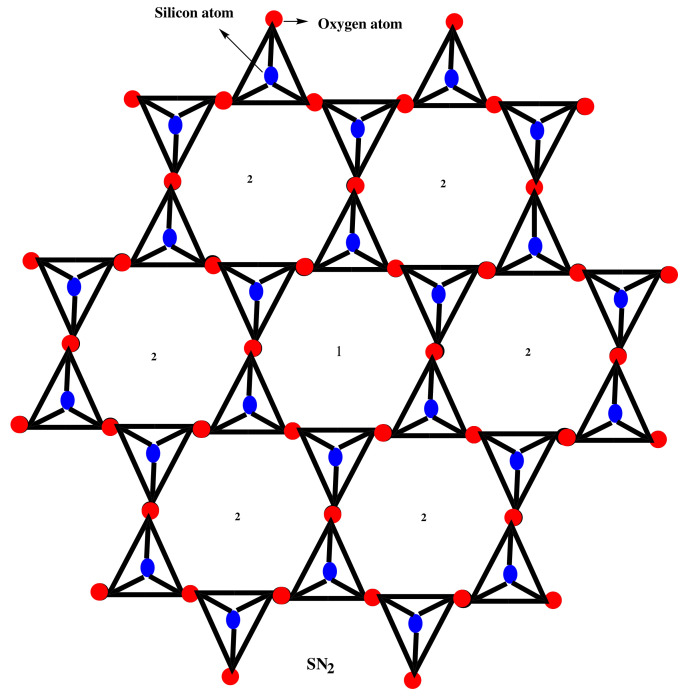
Silicate network of dimension 2.

In this section, we shall compute temperature indices for silicate networks. Metal oxide or metal carbonates are fused with sand to form silicate networks. The basic unit of silicates is the tetrahedron $SiO_{4}$; this tetrahedron is found in almost all silicates. The sides of the tetrahedron $SiO_{4}$ represent oxygen atoms, while the middle represents silicon atoms from a chemical perspective. Figure 1 depicts a tetrahedron of $SiO_{4}$ in a silicate network $SN_{P}$, where *p* is the number of hexagons between the center and the boundary of $SN_{P}$. A silicate sheet network is a collection of $SiO_{4}$ linked to other rings in a two-dimensional plane by shared oxygen atoms, resulting in a sheet-like structure, as shown in Figure 1.

It can be seen in silicate network $SN_{P}$ (see Figure 1) that silicon atoms and corner atoms (lying on $SiO_{4}$ tetrahedrons in each ring) have a degree of 3, whereas all other atoms have a degree of 6. The number of atoms of degree 3 and degree 6 are $6p^{2}+6p$ and $9p^{2}-3p$, respectively. Thus, the total number of atoms and total number of atom bonds is shown in Equation (10).

(10) $$|V\left(SN_{P}\right)|\;=\;3\left(5p^{2}+1\right)\;\;and\;\;\left|E\left(SN_{P}\right)\right|=36p^{2}$$

According to the degree of the atoms, there are three types of atom bonds in $SN_{P}$: (3,3), (3,6) and (6,6). The atom bond partition of $SN_{P}$ can be shown as:

$$\begin{array}{ccc} E_{\left(2,2\right)} & = & \{e=u∼v,\forall \;u,v\in V\left(SN_{P}\right)|d_{u}=3,d_{v}=3\},\;\left|E_{\left(3,3\right)}\right|=6p\\ E_{\left(2,3\right)} & = & \{e=u∼v,\forall \;u,v\in V\left(SN_{P}\right)|d_{u}=3,d_{v}=6\},\;\left|E_{\left(3,6\right)}\right|=6\left(3p^{2}+1\right)\\ E_{\left(3,3\right)} & = & \{e=u∼v,\forall \;u,v\in V\left(SN_{P}\right)|d_{u}=6,d_{v}=6\},\;\left|E_{\left(6,6\right)}\right|=6\left(3p^{2}-2p\right). \end{array}$$

Using Equation (1) and above partition of $SN_{P}$, it can be seen that there are three types of edges based on the temperature of end vertices of each edge, as given in Table 1.

**Theorem** **1**. *Let $SN_{P}$ be a silicate network. Then, the first temperature index is $\frac{12}{5p}+6\left(3p^{2}+1\right)\frac{15p^{2}-1}{5p^{2}\left(5P^{2}-1\right)}+\frac{24p(3p-2)}{5p^{2}-1}$.*

**Proof.**  Using the atom bond partition from Table 1 in the formula of the first temperature index (2), we obtain

$$\begin{array}{ccc} T_{1}\left(SN_{P}\right) & = & \sum_{E_{\left(3,3\right)}}\left(T_{3}+T_{3}\right)+\sum_{E_{\left(3,6\right)}}\left(T_{3}+T_{6}\right)+\sum_{E_{\left(6,6\right)}}\left(T_{6}+T_{6}\right)\\ & = & 6p\left[\frac{3}{3(5p^{2}+1)-3}+\frac{3}{3(5p^{2}+1)-3}\right]+6\left(3p^{2}+1\right)\left[\frac{3}{3(5p^{2}+1)-3}+\frac{6}{3(5p^{2}+1)-6}\right]\\ & + & 6\left(3p^{2}-2p\right)\left[\frac{6}{3(5p^{2}+1)-6}+\frac{6}{3(5p^{2}+1)-6}\right] \end{array}$$

After simplification, we obtain

(11) $$\begin{array}{c} T_{1}\left(SN_{P}\right)=\frac{12}{5p}+6\left(3p^{2}+1\right)\frac{15p^{2}-1}{5p^{2}\left(5P^{2}-1\right)}+\frac{24p(3p-2)}{5p^{2}-1}. \end{array}$$

□

**Theorem** **2**. *Let $SN_{P}$ be a silicate network. Then, the second temperature index is $\frac{6}{25p^{3}}+\frac{12(3p^{2}+1)}{5p^{2}\left(5P^{2}-1\right)}+\frac{24p(3p-2)}{{\left(5p^{2}-1\right)}^{2}}$.*

**Proof.**  Using the atom bond partition from Table 1 in the formula of the second temperature index (3), we obtain

$$\begin{array}{ccc} T_{2}\left(SN_{P}\right) & = & \sum_{E_{\left(3,3\right)}}\left(T_{3}\times T_{3}\right)+\sum_{E_{\left(3,6\right)}}\left(T_{3}\times T_{6}\right)+\sum_{E_{\left(6,6\right)}}\left(T_{6}\times T_{6}\right)\\ & = & 6p\left[\frac{3}{3(5p^{2}+1)-3}\times \frac{3}{3(5p^{2}+1)-3}\right]+6\left(3p^{2}+1\right)\left[\frac{3}{3(5p^{2}+1)-3}\times \frac{6}{3(5p^{2}+1)-6}\right]\\ & + & 6\left(3p^{2}-2p\right)\left[\frac{6}{3(5p^{2}+1)-6}\times \frac{6}{3(5p^{2}+1)-6}\right] \end{array}$$

After simplification, we obtain

(12) $$\begin{array}{c} T_{2}\left(SN_{P}\right)=\frac{6}{25p^{3}}+\frac{12(3p^{2}+1)}{5p^{2}\left(5P^{2}-1\right)}+\frac{24p(3p-2)}{{\left(5p^{2}-1\right)}^{2}}. \end{array}$$

□

**Theorem** **3**. *Let $SN_{P}$ be a silicate network. Then, the first hyper temperature index is $\frac{24}{25p^{3}}+6\left(3p^{2}+1\right)\frac{{\left(15p^{2}-1\right)}^{2}}{25p^{4}{\left(5P^{2}-1\right)}^{2}}+\frac{96p(3p-2)}{{\left(5p^{2}-1\right)}^{2}}$.*

**Proof.**  Using the atom bond partition from Table 1 in the formula of the first hyper temperature index (4), we obtain

$$\begin{array}{ccc} HT_{1}\left(SN_{P}\right) & = & \sum_{E_{\left(3,3\right)}}{\left(T_{3}+T_{3}\right)}^{2}+\sum_{E_{\left(3,6\right)}}{\left(T_{3}+T_{6}\right)}^{2}+\sum_{E_{\left(6,6\right)}}{\left(T_{6}+T_{6}\right)}^{2}\\ & = & 6p{\left[\frac{3}{3(5p^{2}+1)-3}+\frac{3}{3(5p^{2}+1)-3}\right]}^{2}+6\left(3p^{2}+1\right){\left[\frac{3}{3(5p^{2}+1)-3}+\frac{6}{3(5p^{2}+1)-6}\right]}^{2}\\ & + & 6\left(3p^{2}-2p\right){\left[\frac{6}{3(5p^{2}+1)-6}+\frac{6}{3(5p^{2}+1)-6}\right]}^{2} \end{array}$$

After simplification, we obtain

(13) $$\begin{array}{c} HT_{1}\left(SN_{P}\right)=\frac{24}{25p^{3}}+6\left(3p^{2}+1\right)\frac{{\left(15p^{2}-1\right)}^{2}}{25p^{4}{\left(5P^{2}-1\right)}^{2}}+\frac{96p(3p-2)}{{\left(5p^{2}-1\right)}^{2}}. \end{array}$$

□

**Theorem** **4**. *Let $SN_{P}$ be a silicate network. Then, the second hyper temperature index is $\frac{6}{625p^{7}}+\frac{24(3p^{2}+1)}{25p^{4}{\left(5P^{2}-1\right)}^{2}}+\frac{96p(3p-2)}{{\left(5p^{2}-1\right)}^{4}}$.*

**Proof.**  Using the atom bond partition from Table 1 in the formula of the second temperature index (5), we obtain

$$\begin{array}{ccc} HT_{2}\left(SN_{P}\right) & = & \sum_{E_{\left(3,3\right)}}{\left(T_{3}\times T_{3}\right)}^{2}+\sum_{E_{\left(3,6\right)}}{\left(T_{3}\times T_{6}\right)}^{2}+\sum_{E_{\left(6,6\right)}}{\left(T_{6}\times T_{6}\right)}^{2}\\ & = & 6p{\left[\frac{3}{3(5p^{2}+1)-3}\times \frac{3}{3(5p^{2}+1)-3}\right]}^{2}\\ & + & 6\left(3p^{2}+1\right){\left[\frac{3}{3(5p^{2}+1)-3}\times \frac{6}{3(5p^{2}+1)-6}\right]}^{2}\\ & + & 6\left(3p^{2}-2p\right){\left[\frac{6}{3(5p^{2}+1)-6}\times \frac{6}{3(5p^{2}+1)-6}\right]}^{2} \end{array}$$

After simplification, we obtain

(14) $$\begin{array}{c} HT_{2}\left(SN_{P}\right)=\frac{6}{625p^{7}}+\frac{24(3p^{2}+1)}{25p^{4}{\left(5P^{2}-1\right)}^{2}}+\frac{96p(3p-2)}{{\left(5p^{2}-1\right)}^{4}}. \end{array}$$

□

**Theorem** **5**. *Let $SN_{P}$ be a silicate network. Then, the sum connectivity temperature index is $3p^{2}\sqrt{10}+\frac{6p\left(3p^{2}+1\right)\sqrt{5(5p^{2}-1)}}{\sqrt{15P^{2}-1}}+3p\left(3p-2\right)\sqrt{\left(5p^{2}-1\right)}$.*

**Proof.**  Using the atom bond partition from Table 1 in the formula of the sum connectivity temperature index (6), we obtain

$$\begin{array}{ccc} ST(SN_{P}) & = & \sum_{E_{\left(3,3\right)}}\frac{1}{\sqrt{\left(T_{3}+T_{3}\right)}}+\sum_{E_{\left(3,6\right)}}\frac{1}{\sqrt{\left(T_{3}+T_{6}\right)}}+\sum_{E_{\left(6,6\right)}}\frac{1}{\sqrt{\left(T_{6}+T_{6}\right)}}\\ & = & \frac{6p}{\sqrt{\left[\frac{3}{3(5p^{2}+1)-3}+\frac{3}{3(5p^{2}+1)-3}\right]}}+\frac{6(3p^{2}+1)}{\sqrt{\left[\frac{3}{3(5p^{2}+1)-3}+\frac{6}{3(5p^{2}+1)-6}\right]}}+\frac{6(3p^{2}-2p)}{\sqrt{\left[\frac{6}{3(5p^{2}+1)-6}+\frac{6}{3(5p^{2}+1)-6}\right]}} \end{array}$$

After simplification, we obtain

(15) $$\begin{array}{c} ST\left(SN_{P}\right)=3p^{2}\sqrt{10}+\frac{6p\left(3p^{2}+1\right)\sqrt{5(5p^{2}-1)}}{\sqrt{15P^{2}-1}}+3p\left(3p-2\right)\sqrt{\left(5p^{2}-1\right)}. \end{array}$$

□

**Theorem** **6**. *Let $SN_{P}$ be a silicate network. Then, the product connectivity temperature index is $30p^{2}+\frac{3p\left(3p^{2}+1\right)\sqrt{2}}{\sqrt{5p^{2}\left(5P^{2}-1\right)}}+3p\left(3p-2\right)\left(5p^{2}-1\right)$.*

**Proof.**  Using the atom bond partition from Table 1 in the formula of the product connectivity temperature index (7), we obtain

$$\begin{array}{ccc} PT(SN_{P}) & = & \sum_{E_{\left(3,3\right)}}\frac{1}{\sqrt{\left(T_{3}\times T_{3}\right)}}+\sum_{E_{\left(3,6\right)}}\frac{1}{\sqrt{\left(T_{3}\times T_{6}\right)}}+\sum_{E_{\left(6,6\right)}}\frac{1}{\sqrt{\left(T_{6}\times T_{6}\right)}}\\ & = & \frac{6p}{\sqrt{\left[\frac{3}{3(5p^{2}+1)-3}\times \frac{3}{3(5p^{2}+1)-3}\right]}}+\frac{6(3p^{2}+1)}{\sqrt{\left[\frac{3}{3(5p^{2}+1)-3}\times \frac{6}{3(5p^{2}+1)-6}\right]}}+\frac{6(3p^{2}-2p)}{\sqrt{\left[\frac{6}{3(5p^{2}+1)-6}\times \frac{6}{3(5p^{2}+1)-6}\right]}} \end{array}$$

After simplification, we obtain

(16) $$\begin{array}{c} PT\left(SN_{P}\right)=30p^{2}+\frac{3p\left(3p^{2}+1\right)\sqrt{2}}{\sqrt{5p^{2}\left(5P^{2}-1\right)}}+3p\left(3p-2\right)\left(5p^{2}-1\right). \end{array}$$

□

**Theorem** **7**. *Let $SN_{P}$ be a silicate network. Then, the reciprocal product temperature index is $\frac{6}{5}+\frac{6\left(3p^{2}+1\right)\sqrt{2}}{\sqrt{5p^{2}\left(5P^{2}-1\right)}}+\frac{12p(3p-2)}{5p^{2}-1}$.*

**Proof.**  Using the atom bond partition from Table 1 in the formula of the second temperature index (8), we obtain

$$\begin{array}{ccc} RPT(SN_{P}) & = & \sum_{E_{\left(3,3\right)}}\sqrt{\left(T_{3}\times T_{3}\right)}+\sum_{E_{\left(3,6\right)}}\sqrt{\left(T_{3}\times T_{6}\right)}+\sum_{E_{\left(6,6\right)}}\sqrt{\left(T_{6}\times T_{6}\right)}\\ & = & 6p\sqrt{\left[\frac{3}{3(5p^{2}+1)-3}\times \frac{3}{3(5p^{2}+1)-3}\right]}\\ & + & 6\left(3p^{2}+1\right)\sqrt{\left[\frac{3}{3(5p^{2}+1)-3}\times \frac{6}{3(5p^{2}+1)-6}\right]}\\ & + & 6\left(3p^{2}-2p\right)\sqrt{\left[\frac{6}{3(5p^{2}+1)-6}\times \frac{6}{3(5p^{2}+1)-6}\right]} \end{array}$$

After simplification, we obtain

(17) $$\begin{array}{c} RPT\left(SN_{P}\right)=\frac{6}{5}+\frac{6\left(3p^{2}+1\right)\sqrt{2}}{\sqrt{5p^{2}\left(5P^{2}-1\right)}}+\frac{12p(3p-2)}{5p^{2}-1}. \end{array}$$

□

**Theorem** **8**. *Let $SN_{P}$ be a silicate network. Then, the F-temperature index is.*

**Proof.**  Using the atom bond partition from Table 1 in the formula of the F-temperature index (9), we obtain

$$\begin{array}{ccc} FT(SN_{P}) & = & \sum_{E_{\left(3,3\right)}}\left(T_{3}^{2}+T_{3}^{2}\right)+\sum_{E_{\left(3,6\right)}}\left(T_{3}^{2}+T_{6}^{2}\right)+\sum_{E_{\left(6,6\right)}}\left(T_{6}^{2}+T_{6}^{2}\right)\\ & = & 6p\left[\{\frac{3}{3(5p^{2}+1)-3}{\}}^{2}+\{\frac{3}{3(5p^{2}+1)-3}{\}}^{2}\right]\\ & + & 6\left(3p^{2}+1\right)\left[\{\frac{3}{3(5p^{2}+1)-3}{\}}^{2}+\{\frac{6}{3(5p^{2}+1)-6}{\}}^{2}\right]\\ & + & 6\left(3p^{2}-2p\right)\left[\{\frac{6}{3(5p^{2}+1)-6}{\}}^{2}+\{\frac{6}{3(5p^{2}+1)-6}{\}}^{2}\right] \end{array}$$

After simplification, we obtain

(18) $$\begin{array}{c} FT\left(SN_{P}\right)=\frac{12}{25p^{3}}+6\left(3p^{2}+1\right)\frac{{\left(15p^{2}-1\right)}^{2}+100p^{4}}{25p^{4}{\left(5P^{2}-1\right)}^{2}}+\frac{48p(3p-2)}{{\left(5p^{2}-1\right)}^{2}}. \end{array}$$

□

### Numerical Comparison of Temperature Indices for $SN_{p}$

In this section, we present a numerical comparison in Table 2 of temperature indices for n=2,3,4,…,15 of silicate network $SN_{p}$.

## 3. Results for Silicate Chain Network $CN_{P}$

**Figure 2:**
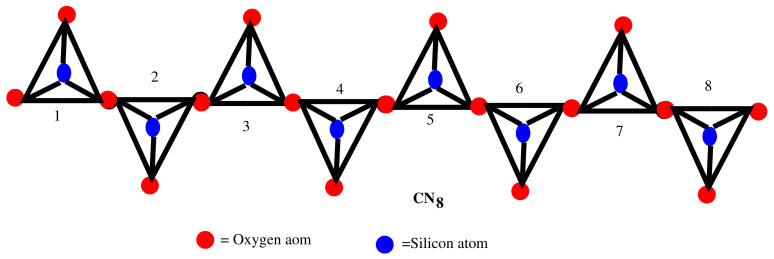
Silicate chain network of dimension 8.

In this section, we will look at a family of silicate chain networks, which is denoted by $CN_{P}$ and is obtained by arranging *p* tetrahedral $SiO_{4}$ linearly, as shown in Figure 2.

It can be seen in silicate chain network $CN_{P}$ (see Figure 2) that the silicon atoms and corner atoms (lying on $SiO_{4}$ tetrahedrons in each ring) have valency 3, where as all other atoms have valency 6. The number of atoms of valency 3 and valency 6 are 2(p+1) and p−1, respectively. Thus, the total number of atoms and total number of atom bonds is shown in Equation (19).

(19) $$|V\left(CN_{P}\right)|=3p+1\;\;and\;\;|E\left(CN_{P}\right)|=6p$$

According to the degree of the atoms, there are three types of atom bonds in $CN_{P}$: (3,3), (3,6) and (6,6). The atom bond partition of $CN_{P}$ is shown as:

$$\begin{array}{ccc} E_{\left(2,2\right)} & = & \{e=u∼v,\forall \;u,v\in V\left(CN_{P}\right)|d_{u}=3,d_{v}=3\},\;\left|E_{\left(3,3\right)}\right|=p+4\\ E_{\left(2,3\right)} & = & \{e=u∼v,\forall \;u,v\in V\left(CN_{P}\right)|d_{u}=3,d_{v}=6\},\;\left|E_{\left(3,6\right)}\right|=2\left(2p-1\right)\\ E_{\left(3,3\right)} & = & \{e=u∼v,\forall \;u,v\in V\left(CN_{P}\right)|d_{u}=6,d_{v}=6\},\;\left|E_{\left(6,6\right)}\right|=p-2. \end{array}$$

Using Equation (1) and above partition of $CN_{P}$, it can be seen that there are three types of edges based on the temperature of end vertices of each edge, as given in Table 3.

**Theorem** **9**. *Let $CN_{P}$ be a silicate chain network. Then, the first temperature index is $\frac{6(p+4)}{3p-2}+\frac{54(2p^{2}-3p+1)}{9p^{2}-21p+10}+\frac{12(p-2)}{3p-5}$.*

**Proof.**  Using the atom bond partition from Table 3 in the formula of the first temperature index (2), we obtain

$$\begin{array}{ccc} T_{1}\left(CN_{P}\right) & = & \sum_{E_{\left(3,3\right)}}\left(T_{3}+T_{3}\right)+\sum_{E_{\left(3,6\right)}}\left(T_{3}+T_{6}\right)+\sum_{E_{\left(6,6\right)}}\left(T_{6}+T_{6}\right)\\ & = & \left(p+4\right)\left[\frac{3}{(3p+1)-3}+\frac{3}{(3p+1)-3}\right]+2\left(2p-1\right)\left[\frac{3}{(3p+1)-3}+\frac{6}{(3p+1)-6}\right]\\ & + & \left(p-2\right)\left[\frac{6}{(3p+1)-6}+\frac{6}{(3p+1)-6}\right] \end{array}$$

After simplification, we obtain

(20) $$\begin{array}{c} T_{1}\left(CN_{P}\right)=\frac{6(p+4)}{3p-2}+\frac{54(2p^{2}-3p+1)}{9p^{2}-21p+10}+\frac{12(p-2)}{3p-5}. \end{array}$$

□

**Theorem** **10**. *Let $CN_{P}$ be a silicate chain network. Then, the second temperature index is $\frac{9(p+4)}{{\left(3p-2\right)}^{2}}+\frac{36(2p-1)}{9p^{2}-21p+10}+\frac{36(p-2)}{{\left(3p-5\right)}^{2}}$.*

**Proof.**  Using the atom bond partition from Table 3 in the formula of the second temperature index (3), we obtain

$$\begin{array}{ccc} T_{2}\left(CN_{P}\right) & = & \sum_{E_{\left(3,3\right)}}\left(T_{3}\times T_{3}\right)+\sum_{E_{\left(3,6\right)}}\left(T_{3}\times T_{6}\right)+\sum_{E_{\left(6,6\right)}}\left(T_{6}\times T_{6}\right)\\ & = & \left(p+4\right)\left[\frac{3}{(3p+1)-3}\times \frac{3}{(3p+1)-3}\right]+2\left(2p-1\right)\left[\frac{3}{(3p+1)-3}\times \frac{6}{(3p+1)-6}\right]\\ & + & \left(p-2\right)\left[\frac{6}{(3p+1)-6}\times \frac{6}{(3p+1)-6}\right] \end{array}$$

After simplification, we obtain

(21) $$\begin{array}{c} T_{2}\left(CN_{P}\right)=\frac{9(p+4)}{{\left(3p-2\right)}^{2}}+\frac{36(2p-1)}{9p^{2}-21p+10}+\frac{36(p-2)}{{\left(3p-5\right)}^{2}}. \end{array}$$

□

**Theorem** **11**. *Let $CN_{P}$ be a silicate chain network. Then, the first hyper temperature index is $\frac{36(p+4)}{{\left(3p-2\right)}^{2}}+\frac{1458\left(2p-1\right){\left(p-1\right)}^{2}}{\left(3p-5\right){\left(3p-2\right)}^{2}}+\frac{144(p-2)}{{\left(3p-5\right)}^{2}}$.*

**Proof.**  Using the atom bond partition from Table 3 in the formula of the first hyper temperature index (4), we obtain

$$\begin{array}{ccc} HT_{1}\left(CN_{P}\right) & = & \sum_{E_{\left(3,3\right)}}{\left(T_{3}+T_{3}\right)}^{2}+\sum_{E_{\left(3,6\right)}}{\left(T_{3}+T_{6}\right)}^{2}+\sum_{E_{\left(6,6\right)}}{\left(T_{6}+T_{6}\right)}^{2}\\ & = & \left(p+4\right){\left[\frac{3}{(3p+1)-3}+\frac{3}{(3p+1)-3}\right]}^{2}+2\left(2p-1\right){\left[\frac{3}{(3p+1)-3}+\frac{6}{(3p+1)-6}\right]}^{2}\\ & + & \left(p-2\right){\left[\frac{6}{(3p+1)-6}+\frac{6}{(3p+1)-6}\right]}^{2} \end{array}$$

After simplification, we obtain

(22) $$\begin{array}{c} HT_{1}\left(CN_{P}\right)=\frac{36(p+4)}{{\left(3p-2\right)}^{2}}+\frac{1458\left(2p-1\right){\left(p-1\right)}^{2}}{\left(3p-5\right){\left(3p-2\right)}^{2}}+\frac{144(p-2)}{{\left(3p-5\right)}^{2}}. \end{array}$$

□

**Theorem** **12**. *Let $CN_{P}$ be a silicate chain network. Then, the second hyper temperature index is $\frac{81(p+4)}{{\left(3p-2\right)}^{4}}+\frac{648(2p-1)}{{\left(3p-5\right)}^{2}{\left(3p-2\right)}^{2}}+\frac{1296(p-2)}{{\left(3p-5\right)}^{4}}$.*

**Proof.**  Using the atom bond partition from Table 3 in the formula of the second temperature index (5), we obtain

$$\begin{array}{ccc} HT_{2}\left(CN_{P}\right) & = & \sum_{E_{\left(3,3\right)}}{\left(T_{3}\times T_{3}\right)}^{2}+\sum_{E_{\left(3,6\right)}}{\left(T_{3}\times T_{6}\right)}^{2}+\sum_{E_{\left(6,6\right)}}{\left(T_{6}\times T_{6}\right)}^{2}\\ & = & \left(p+4\right){\left[\frac{3}{(3p+1)-3}\times \frac{3}{(3p+1)-3}\right]}^{2}+2\left(2p-1\right){\left[\frac{3}{(3p+1)-3}\times \frac{6}{(3p+1)-6}\right]}^{2}\\ & + & \left(p-2\right){\left[\frac{6}{(3p+1)-6}\times \frac{6}{(3p+1)-6}\right]}^{2} \end{array}$$

After simplification, we obtain

(23) $$\begin{array}{c} HT_{2}\left(CN_{P}\right)=\frac{81(p+4)}{{\left(3p-2\right)}^{4}}+\frac{648(2p-1)}{{\left(3p-5\right)}^{2}{\left(3p-2\right)}^{2}}+\frac{1296(p-2)}{{\left(3p-5\right)}^{4}}. \end{array}$$

□

**Theorem** **13**. *Let $CN_{P}$ be a silicate chain network. Then the sum connectivity temperature index is $\frac{\left(p+4\right)\sqrt{3p-2}}{\sqrt{6}}+\frac{2\left(2p-1\right)\sqrt{9p^{2}-21p+10}}{\sqrt{27(p-1)}}+\frac{\left(p-2\right)\sqrt{3p-5}}{\sqrt{12}}$.*

**Proof.**  Using the atom bond partition from Table 3 in the formula of the sum connectivity temperature index (6), we obtain

$$\begin{array}{ccc} ST(CN_{P}) & = & \sum_{E_{\left(3,3\right)}}\frac{1}{\sqrt{\left(T_{3}+T_{3}\right)}}+\sum_{E_{\left(3,6\right)}}\frac{1}{\sqrt{\left(T_{3}+T_{6}\right)}}+\sum_{E_{\left(6,6\right)}}\frac{1}{\sqrt{\left(T_{6}+T_{6}\right)}}\\ & = & \left(p+4\right)\frac{1}{\sqrt{\left[\frac{3}{(3p+1)-3}+\frac{3}{(3p+1)-3}\right]}}+2\left(2p-1\right)\frac{1}{\sqrt{\left[\frac{3}{(3p+1)-3}+\frac{6}{(3p+1)-6}\right]}}\\ & + & \left(p-2\right)\frac{1}{\sqrt{\left[\frac{6}{(3p+1)-6}+\frac{6}{(3p+1)-6}\right]}} \end{array}$$

After simplification, we obtain

(24) $$\begin{array}{c} ST\left(CN_{P}\right)=\frac{\left(p+4\right)\sqrt{3p-2}}{\sqrt{6}}+\frac{2\left(2p-1\right)\sqrt{9p^{2}-21p+10}}{\sqrt{27(p-1)}}+\frac{\left(p-2\right)\sqrt{3p-5}}{\sqrt{12}}. \end{array}$$

□

**Theorem** **14**. *Let $CN_{P}$ be a silicate network. Then, the product connectivity temperature index is $\frac{3p^{2}+10p-8}{3}+\frac{2\left(2p-1\right)\sqrt{9p^{2}-21p+10}}{\sqrt{18}}+\frac{3p^{2}-11p+10}{6}$.*

**Proof.**  Using the atom bond partition from Table 3 in the formula of the product connectivity temperature index (7), we obtain

$$\begin{array}{ccc} PT(CN_{P}) & = & \sum_{E_{\left(3,3\right)}}\frac{1}{\sqrt{\left(T_{3}\times T_{3}\right)}}+\sum_{E_{\left(3,6\right)}}\frac{1}{\sqrt{\left(T_{3}\times T_{6}\right)}}+\sum_{E_{\left(6,6\right)}}\frac{1}{\sqrt{\left(T_{6}\times T_{6}\right)}}\\ & = & \left(p+4\right)\frac{1}{\sqrt{\left[\frac{3}{(3p+1)-3}\times \frac{3}{(3p+1)-3}\right]}}+2\left(2p-1\right)\frac{1}{\sqrt{\left[\frac{3}{(3p+1)-3}\times \frac{6}{(3p+1)-6}\right]}}\\ & + & \left(p-2\right)\frac{1}{\sqrt{\left[\frac{6}{(3p+1)-6}\times \frac{6}{(3p+1)-6}\right]}} \end{array}$$

After simplification, we obtain

(25) $$\begin{array}{c} PT\left(CN_{P}\right)=\frac{3p^{2}+10p-8}{3}+\frac{2\left(2p-1\right)\sqrt{9p^{2}-21p+10}}{\sqrt{18}}+\frac{3p^{2}-11p+10}{6}. \end{array}$$

□

**Theorem** **15**. *Let $CN_{P}$ be a silicate chain network. Then, the reciprocal product temperature index is $\frac{3(p+4)}{3p-2}+\frac{6\left(2p-1\right)\sqrt{2}}{\sqrt{9p^{2}-21p+10}}+\frac{6(p-2)}{3p-5}$.*

**Proof.**  Using the atom bond partition from Table 3 in the formula of the second temperature index (8), we obtain

$$\begin{array}{ccc} RPT(CN_{P}) & = & \sum_{E_{\left(3,3\right)}}\sqrt{\left(T_{3}\times T_{3}\right)}+\sum_{E_{\left(3,6\right)}}\sqrt{\left(T_{3}\times T_{6}\right)}+\sum_{E_{\left(6,6\right)}}\sqrt{\left(T_{6}\times T_{6}\right)}\\ & = & \left(p+4\right)\sqrt{\left[\frac{3}{(3p+1)-3}\times \frac{3}{(3p+1)-3}\right]}+2\left(2p-1\right)\sqrt{\left[\frac{3}{(3p+1)-3}\times \frac{6}{(3p+1)-6}\right]}\\ & + & \left(p-2\right)\sqrt{\left[\frac{6}{(3p+1)-6}\times \frac{6}{(3p+1)-6}\right]} \end{array}$$

After simplification, we obtain

(26) $$\begin{array}{c} RPT\left(CN_{P}\right)=\frac{3(p+4)}{3p-2}+\frac{6\left(2p-1\right)\sqrt{2}}{\sqrt{9p^{2}-21p+10}}+\frac{6(p-2)}{3p-5}. \end{array}$$

□

**Theorem** **16**. *Let $CN_{P}$ be a silicate network. Then, the F-temperature index is $\frac{18(p+4)}{{\left(3p-2\right)}^{2}}$$+\frac{6\left(2p-1\right)\left(135p^{2}-243p+123\right)}{{\left(3p-2\right)}^{2}{\left(3p-5\right)}^{2}}+\frac{72(p-2)}{{\left(3p-5\right)}^{2}}$.*

**Proof.**  Using the atom bond partition from Table 3 in the formula of the F-temperature index (9), we obtain

$$\begin{array}{ccc} FT(CN_{P}) & = & \sum_{E_{\left(3,3\right)}}\left(T_{3}^{2}+T_{3}^{2}\right)+\sum_{E_{\left(3,6\right)}}\left(T_{3}^{2}+T_{6}^{2}\right)+\sum_{E_{\left(6,6\right)}}\left(T_{6}^{2}+T_{6}^{2}\right)\\ & = & \left(p+4\right)\left[\{\frac{3}{(3p+1)-3}{\}}^{2}+\{\frac{3}{(3p+1)-3}{\}}^{2}\right]\\ & + & 2\left(2p-1\right)\left[\{\frac{3}{(3p+1)-3}{\}}^{2}+\{\frac{6}{(3p+1)-6}{\}}^{2}\right]\\ & + & \left(p-2\right)\left[\{\frac{6}{(3p+1)-6}{\}}^{2}+\{\frac{6}{(3p+1)-6}{\}}^{2}\right] \end{array}$$

After simplification, we obtain

(27) $$\begin{array}{c} FT\left(CN_{P}\right)=\frac{18(p+4)}{{\left(3p-2\right)}^{2}}+\frac{6\left(2p-1\right)\left(135p^{2}-243p+123\right)}{{\left(3p-2\right)}^{2}{\left(3p-5\right)}^{2}}+\frac{72(p-2)}{{\left(3p-5\right)}^{2}}. \end{array}$$

□

### Numerical Comparison of Temperature Indices for $CN_{p}$

In this section, we present a numerical comparison of temperature indices for n=2,3,4,…,15 of silicate chain network $CN_{p}$ (Table 4).

## 4. Graphical Comparison of Temperature Indices and Conclusion

**Figure 3:**
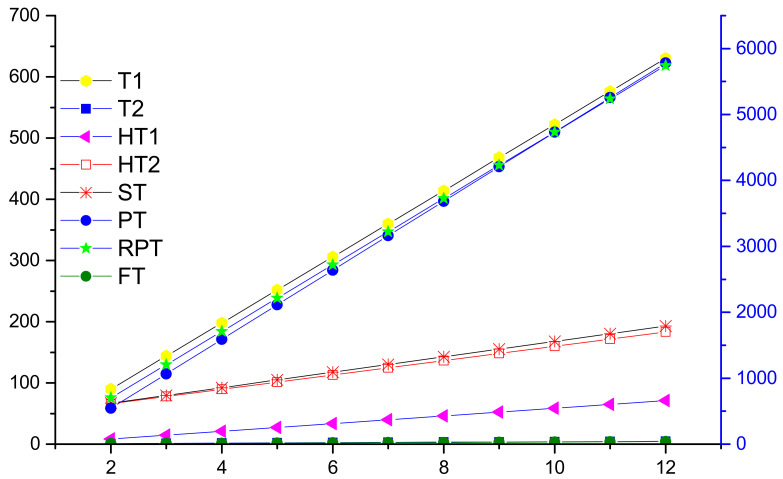
2D graphical comparison of temperature indices.

Here, we try to show the variations of temperature indices in a 2D comparison graph; see Figure 3. The sum connectivity temperature index ST and the product connectivity temperature index PT gradually increase; however, the values of $T_{1}$, $T_{2}$, $HT_{1}$, $HT_{2}$, RPT, and FT rapidly decrease whenever the number of $SiO_{4}$ increases in the silicate and silicate chain network.

In QSPR/QSAR research, topological indices including the Zagreb index, Randic index, and atom bond connectivity index are utilised to predict chemical compound bioactivity. We propose computing the first temperature index, second temperature index, first hyper temperature index, second hyper temperature index, sum temperature index, product temperature, reciprocal product temperature index, and F-temperature index of silicate networks and silicate chain networks, which correlates well with entropy, the acentric factor, the enthalpy of vaporisation, and the standard enthalpy of vaporisation.

## Figures and Tables

**Table 1:** Atom bond partition of $SN_{P}$ based on the valency of each atom of $SiO_{4}$.

$\left(T_{u},T_{v}\right)$	$\left(\frac{3}{3(5p^{2}+1)-3},\frac{3}{3(5p^{2}+1)-3}\right)$	$\left(\frac{3}{3(5p^{2}+1)-3},\frac{6}{3(5p^{2}+1)-6}\right)$	$\left(\frac{6}{3(5p^{2}+1)-6},\frac{6}{3(5p^{2}+1)-6}\right)$
	6p	$6(3p^{2}+1)$	$6(3p^{2}-2p)$

**Table 2:** Temperature indices of silicate network $SN_{p}$ for p≥2.

*p*	$T_{1}$	$T_{2}$	${\mathrm{HT}}_{1}$	${\mathrm{HT}}_{2}$	ST	PT	RPT	FT
2	23.42	0.97	2.44	0.0092538	340.51	581.66	17.57	37.06
3	23.62	0.44	1.16	0.0010648	1149.06	3050	22.05	34.49
4	23.87	0.25	0.68	0.0002780	2730.22	9970.46	28.20	33.58
5	24.06	0.16	0.44	0.00011078	5324.19	24,942.95	33.39	33.16
6	24.21	0.11	0.31	0.00005594	9251.16	52,647.46	38.55	32.93
7	24.33	0.08	0.23	0.00003258	14,711.32	98,843.98	43.70	32.79
8	24.42	0.06	0.18	0.00002082	21,984.83	170,372.50	48.83	32.70
9	24.50	0.05	0.14	0.000014198	31,331.89	275,153.03	53.95	32.64
10	24.56	0.04	0.12	0.000010149	43,012.66	422,185.57	59.07	32.59
11	24.61	0.03	0.10	0.0000075	57,287.32	621,550.10	64.18	32.56
12	24.65	0.03	0.08	0.000057	74,416.05	884,406.64	69.29	32.53

**Table 3:** Atom bond partition of $CN_{P}$ based on the valency of each atom of SiO4.

$\left(T_{u},T_{v}\right)$	$\left(\frac{3}{(3p+1)-3},\frac{3}{(3p+1)-3}\right)$	$\left(\frac{3}{(3p+1)-3},\frac{6}{(3p+1)-6}\right)$	$\left(\frac{6}{(3p+1)-6},\frac{6}{(3p+1)-6}\right)$
Frequency	p+4	2(2p−1)	p−2

**Table 4:** Temperature indices of silicate chain network $CN_{p}$ for p≥2.

*p*	$T_{1}$	$T_{2}$	${\mathrm{HT}}_{1}$	${\mathrm{HT}}_{2}$	ST	PT	RPT	FT
2	49.5	30.38	286.88	123.40	6.55	10.83	17.23	205.88
3	28.29	9.96	162.92	21.83	14.05	29.47	12.53	30.38
4	24.43	5.79	139.98	7.62	23.18	56.61	11.21	15.62
5	22.71	4.05	130.47	3.87	33.72	92.37	10.57	10.41
6	21.72	3.11	125.29	2.34	45.50	147.53	10.19	7.78
7	21.08	2.52	122.04	1.56	58.41	189.85	9.94	6.67
8	20.63	2.11	119.82	1.12	72.36	251.57	9.76	6.21
9	20.29	1.82	118.20	0.84	87.27	321.94	9.62	4.41
10	20.03	1.60	116.97	0.66	103.09	400.97	9.51	3.85
11	19.82	1.42	116	0.53	119.77	488.66	9.43	3.41
12	19.66	1.28	115.22	0.43	137.26	585.46	9.36	3.07

## Data Availability

No data were used to support this study.

## Sample Availability

Not available.
